# Chidamide epigenetically represses autophagy and exerts cooperative antimyeloma activity with bortezomib

**DOI:** 10.1038/s41419-020-2414-3

**Published:** 2020-04-27

**Authors:** Li Xu, Juan Feng, Hailong Tang, Ying Dong, Mimi Shu, Xiequn Chen

**Affiliations:** 1 Department of Hematology, Xijing Hospital, Fourth Military Medical University, Xian, 710032 Shaanxi China; 2 Hematology & Oncology Center, Affiliated Hospital of Northwest University & Xian No.3 Hospital, Xian, 710082 Shaanxi China; 3 Institute of Hematology, School of Medicine, Northwest University, Xian, 710069 Shaanxi China

**Keywords:** Myeloma, Autophagy, Epigenetics, Chemotherapy

## Abstract

Autophagy and ubiquitin proteasome system are two distinct and cooperative proteolytic pathways. The dual-pathway suppression represents a promising therapeutic strategy for multiple myeloma. Chidamide is a novel benzamide inhibitor of histone deacetylase, and shows potent antimyeloma activity. Here, we revealed the autophagy-suppressive role of chidamide in myeloma cells. We then demonstrated that chidamide treatment markedly downregulated histone deacetylase SIRT1, and simultaneously resulted in dose-dependent upregulation of acetyltransferase hMOF and histone methyltransferase EZH2, which contributed to an increase in global levels of histone H4 lysine 16 acetylation (H4k16ac) and histone H3 lysine 27 trimethylation (H3k27me3). We next confirmed concomitant upregulation of H4k16ac and H3k27me3 in the same promoter regions of the autophagy-related gene LC3B, reinforcing the specific roles for H4k16ac and H3k27me3 in mediating chidamide-induced transcriptional repression of LC3B. Finally, we provided experimental evidence that co-treatment with chidamide and proteasome inhibitor bortezomib induced clear synergistic cytotoxicity against MM cells, which was associated with increased accumulation of ubiquitinated proteins and excessive endoplasmic reticulum stress or dysregulated unfolded protein response. Our results altogether suggest that chidamide cooperatively potentiates antimyeloma activity of bortezomib, at least in part, by epigenetically repressing autophagic degradation of ubiquitinated proteins.

## Introduction

Autophagic process leads to the autophagosome-dependent lysosomal degradation of abnormal cytoplasmic components, which involves a series of dynamic membrane remodeling mediated by a core set of autophagy-related (ATG) proteins, such as LC3B^1–3^. Recently, autophagy induction was coupled to epigenetic chromatin changes, especially the amounts of lysine 16 on histone H4 acetylation (H4K16ac) and lysine 27 on histone H3 trimethylation (H3K27me3)^4–9^. Acetylation at H4K16 is positively and negatively regulated by histone acetyltransferase hMOF and its counterpart, histone deacetylase (HDAC) SIRT1^10,11^. Histone methyltransferase EZH2 is primarily responsible for trimethylation at H3K27^12,13^. Recently, these histone-modifying enzymes (hMOF, SIRT1, and EZH2) have emerged as critical regulators of autophagy. Accordingly, H4K16ac and H3K27me3 histone modifications are also reported to be necessary for transcriptional regulation of ATG genes^4–9^.

Multiple myeloma (MM) is a malignancy of antibody-producing plasma cells. MM cells almost always produce large quantities of misfolded immunoglobulins (which account for ~30% of newly synthesized proteins)^14,15^. Accumulation of misfolded proteins in endoplasmic reticulum (ER) (also termed ER stress) subsequently induces the unfolded protein response (UPR), an adaptive signaling pathway that evolved to restore ER homeostasis^15,16^. However, irresolvable or persistent ER stress is able to perturb UPR signaling and trigger apoptosis^17–23^. For cellular proteostasis and survival, MM cells must remove these abnormal proteins via two distinct and cooperative proteolytic pathways, i.e., ubiquitin proteasome system (UPS) and autophagy^14,15^. However, UPS inhibition-induced proteotoxic stress effectively activates autophagic degradation^14,16^, which, in turn, compromises antimyeloma efficacy of proteasome inhibitor (such as bortezomib). Thus, concurrent inhibition of autophagy and proteasome represents a promising strategy for the treatment of MM.

Chidamide is a novel benzamide inhibitor of HDAC, which is independently produced in China^24–26^. Recently, HDAC inhibitors (such as chidamide) were reported to induce DNA damage and apoptotic cell death in a variety of cancers, including MM^27–32^. Moreover, clinical studies provided compelling evidence that HDAC inhibitor panobinostat has cooperative antimyeloma activity when combined with bortezomib^33–35^. Nonetheless, whether and how chidamide and bortezomib act cooperatively in killing myeloma cells remains enigmatic. To address this issue, we characterized the autophagy-suppressive role of chidamide, and then investigated the effects of chidamide on expressions of chromatin-modifying enzymes (hMOF, SIRT1, and EZH2), and associated histone modifications (H4k16ac and H3k27me3). We further determined the epigenetic mechanisms for chidamide-induced autophagy inhibition. Finally, we provided experimental evidence favoring the crucial role of excessive ER stress or dysregulated UPR signaling in mediating cooperative antimyeloma activity of chidamide and bortezomib.

## Materials and methods

### Cell culture and reagents

Human MM cell lines H929 and RPMI8226 were from the American Type Culture Collection (ATCC, Manassas, VA, USA) and verified by STR profiling. Cells were cultured in RPMI 1640 medium supplemented with 10% fetal bovine serum (FCS) in an incubator at 37 °C with 5% CO_2_. Mycoplasma-negative cells were used for subsequent experiments. Reagents were purchased from Sigma-Aldrich unless otherwise specified. Chidamide was a generous gift from Chipscreen Company (Shenzhen, China). Bortezomib (as a pure substance) was from Millennium Pharmaceuticals. Antibodies against LC3B (catalog no. ab51520), SIRT1 (sirtuin1, catalog no. ab32441), hMOF (also called KAT8 or MYST1, catalog no. AP1144b), EZH2 (enhancer of zeste homology 2, catalog no. ab191080), H3 (histone H3, catalog no. ab1791), XBP-1 (X-box-binding protein-1, catalog no. ab109221), Ubiquitin (catalog no. ab137025), CHOP (CCAAT/enhancer-binding protein–homologous protein, catalog no. 179823), BiP (immunoglobulin-binding protein, catalog no. 21985), and GAPDH (glyceraldehyde-3-phosphate dehydrogenase, catalog no. CW0101) were from Abcam (Cambridge, USA). Antibodies against H4K16ac (catalog no. 07-329), H3K27me3 (catalog no. 07-449), and RNA Pol II (RNA Polymerase II) were from Millipore (Temecula, USA). Antibodies against cleaved caspase-3 (catalog no. 9664) and Ki-67 (catalog no. M24001-2) were obtained from Cell Signaling Technology (Boston, USA) and Dako (Glostrup, Denmark), respectively.

### Cell viability and apoptosis assays

Cell viability was assessed by 3-(4,5-dimethylthiazol-2-yl)-2,5-diphenyl tetrazolium (MTT) assay. Apoptotic cell death was evaluated by flow cytometry via annexin V–fluorescein isothiocyanate (FITC) and propidium iodide (PI) double staining using annexin V-FITC detection kit (BD PharMingen, San Diego, USA). Cells with annexin V^+^/PI^−^ or annexin V^+^/PI^+^ were defined as apoptotic cells.

### Measurement of cell proliferation

Cell proliferation was measured by quantifying the cells synthesizing DNA in the S phase of the cell cycle via 5-bromo-2-deoxyuridine (BrdU)-FITC flow kit (BD PharMingen). Cells were seeded in 12-well plates (1 × 10^5^ cells per well) and incubated with 10 µg/ml BrdU for 1 h. BrdU uptake was detected by flow cytometry according to the manufacturer’s protocol.

### Soft-agar clonogenic assay

Colony-formation assay was performed as previously described^36^. Briefly, myeloma cells were suspended in 0.3% agar in RPMI 1640 medium containing 10% FCS, and plated on solidified agar (0.5%) in 24-well plates (1 × 10^4^ cells per well). The plates were incubated for 14 days at 37 °C in 5% CO_2_. The number of colonies was scored using an inverted microscope (Leica, Germany).

### Combination index analysis

Cells were treated for 48 h with different doses of chidamide and bortezomib in monotherapy or in combinations. Two different dose combinations were explored for each combination with a constant ratio between them. The following doses of chidamide and bortezomib (both of them in nM) were used: 150:5; 300:10, and 600:20, respectively. The potency of drug combination was quantified based on the Chou–Talalay equation using CompuSyn software version 1.0 (Chou TC and Martin N. Paramus, NJ, 2005), which calculates a combination index (CI) for each combination point. Based on the resulting values, a plot of CI values over a range of fractions affected (Fa–CI plot) was produced, which quantitatively depicts the additive effect (CI = 1), synergism (CI < 1), and antagonism (CI > 1).

### Quantitative reverse transcriptase polymerase chain reaction (qRT-PCR)

The total RNA was extracted from cells using Trizol reagent (QIAGEN, Shanghai, China). Based on the manufacturer’s instructions, first-strand cDNA synthesis from total RNA was carried out with the RevertAid Kit (Thermo Scientific, Waltham, USA); quantitative PCR (qPCR) was performed using UltraSYBR Mixture kit (CWBIO, Beijing, China). The primer sequences for LC3B, SIRT1, and hMOF were synthesized based on corresponding publications^7,37–39^. GAPDH was used as internal control. Relative expression levels of target mRNA were determined by real-time qRT-PCR in a BioRad Mini device.

### Chromatin immunoprecipitation (ChIP)

ChIP was performed in myeloma cells using EZ ChIP kit (Millipore) according to the manufacturer’s instructions. Briefly, cells were treated with 1% formaldehyde to cross-link the DNA–protein complex. After lysing, DNA was sheared to fragments of 500–700 bp by sonication (BioRad). Pre-cleared chromatin was immunoprecipitated with antibodies against normal rabbit IgG, RNA Pol II, H4K16ac, and H3K27me3. After reversing the formaldehyde cross-linking, DNA was isolated for qPCR with specific primers flanking different regions of LC3B and GAPDH promoters (−144/+22). Histone modifications or GAPDH associated with promoters was assessed by using real-time qPCR. LC3B promoter primers include^7^ region −521/−605, forward 5′-TCTTACAGCCACCAGGAGAGTT-3′, reverse 5′-TTTGTCCCGAGCCTTCATTCTG-3′; region +114/+256, forward 5′-AGGAGATACAAGGGAAGTGGCT-3′, reverse 5′-TTGAAGGTCTTCTCCGACGGCAT-3′. GAPDH promoter primers (Millipore) were used as negative control.

### Immunoblotting

Cells were lysed in lysis buffer as reported previously^3^. Cleared lysates (25–30 μg) were subjected to 10–18% SDS-polyacrylamide gel and transferred onto PVDF (polyvinylidene difluoride) membranes (Millipore). After blocking, filters were incubated with the antibodies indicated in the figures. The bound antibodies were visualized by chemiluminescence using SuperSignal reagent (Pierce).

### Transmission electron microscopy

Electron microscopy analysis was performed as reported previously^3^. Briefly, they were fixed in 2.5% glutaraldehyde in 0.1 mol/L phosphate-buffered saline (PBS) for 1 h at 4 °C. The cells were washed and fixed with 1% osmium tetroxide in 0.1 mol/L PBS for 1 h at 4 °C. Ultrathin sections were prepared, stained with uranyl acetate and lead citrate, and then examined with a Tecnai G2 Spirit 120 KV transmission electron microscope (Thermo Fisher).

### Human plasmacytoma xenograft models

Approval of these studies was provided by the Institutional Animal Care and Use Committee at the Fourth Military Medical University. Female BALB/c nude mice aged 4–6 weeks were from Vital River Laboratory Animal Technology Company (Beijing, China). Mice were bred and kept in a standard animal facility. To increase the rate of tumorigenesis, mice were irradiated for 5 min with cobalt 60 at a dose of 3 Gy. Mice were then subcutaneously injected in the flanks with H929 cells (3 × 10^7^ per mouse) or RPMI8226 cells (1 × 10^7^ per mouse) in 75 µl of serum-free RPMI 1640 medium with 75 µl of Matrigel (BD PharMingen). Approximately 2 weeks after injection, mice were randomized into four groups (*n* = 5 per group) and treated intraperitoneally with triweekly injections of chidamide (10 mg/kg) for 3 consecutive weeks, intravenously with biweekly injections of bortezomib (0.5 mg/kg) for 3 consecutive weeks, or a combination with the same dosing regimen used for the individual drugs. The control group received PBS only at the same schedule as the combination group. Tumor size was measured and calculated as previously described^3^. All animals were sacrificed individually by CO_2_ asphyxiation. TUNEL assay^3^, cleaved caspase-3 expression, and hematoxylin and eosin (H&E) staining were used for in situ detection of apoptosis in tumors. The expression levels of proliferative antigen Ki-67 and cleaved caspase-3 were assessed by immunohistochemical staining.

### Statistical analysis

Each experiment was repeated at least three times unless stated otherwise. All data were normally distributed and presented as mean ± SD. The variance was similar between the groups that are being statistically compared. No samples or mice were excluded from the analysis as outliers. Differences between two data sets (i.e., treated group vs. control) were analyzed using two-tailed Student’s *t* test. Statistical analysis was conducted using SPSS version 24.0 software. Probability values of <0.05 were considered statistically significant. Mice were randomly allocated to groups using the random number table method. Blinding and sample size estimation tests were not done for our animal studies.

## Results

### Chidamide inhibits autophagy by targeting autophagosome and LC3B

**Fig. 1:**
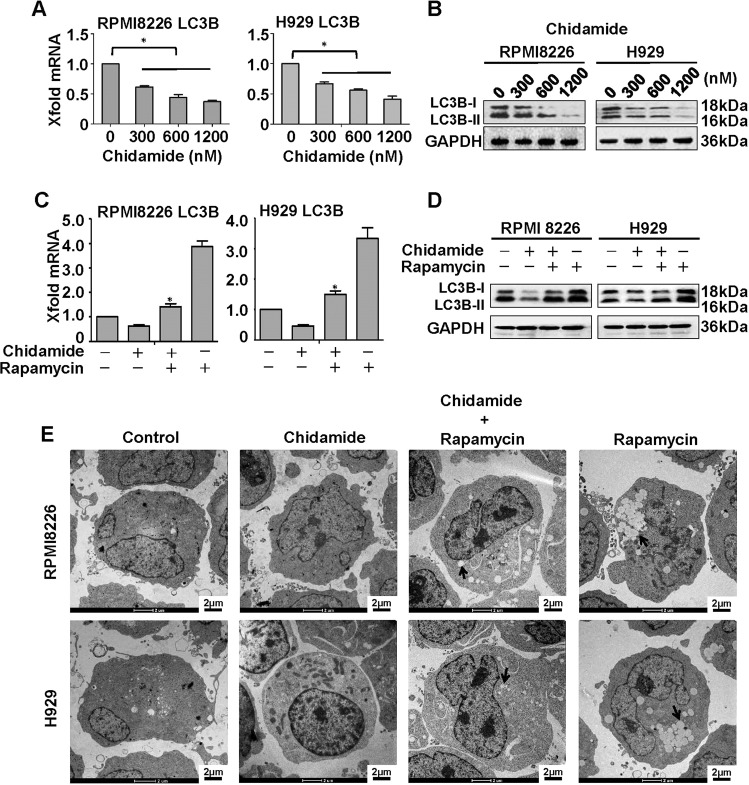
Effects of chidamide alone or in combination with rapamycin on LC3B expression and autophagosome formation in MM cells. RPMI8226 and H929 cells were treated for 48 h with various concentrations of chidamide (**a**, **b**) or with 300 nM chidamide in the presence or absence of 200 nM rapamycin (**c**, **d**). Treatment with rapamycin alone served as a positive control for autophagy induction. Relative LC3B mRNA levels were detected by using quantitative RT-PCR. Mean ± SD of three independent experiments. *P < 0.05, compared with the single-agent groups or treatment-naive control. LC3B protein levels were determined by immunoblotting as indicated. GAPDH was used as a control for protein loading. **e** Electron microscopy pictures were taken. Blots or micrographs shown are representative of three independent experiments. Autophagy vesicles are denoted by arrows. Scale bars: 2 μm. Original magnification, ×6000.

During autophagy, ATG protein LC3B is induced and processed to a cytosolic unlipidated LC3B-I (18 kDa), and then converted to a lipidated LC3B-II (16 kDa) that stably attached to the membrane of autophagic vacuoles (i.e., autophagosomes or autolysosomes). Thus, autophagic response can be identified biochemically (by observing LC3B generation or conversion) and morphologically (by examining the formation of autophagic vacuoles). For these purposes, H929 or RPMI8226 cells were exposed for 24 h to various concentrations of chidamide, and then analyzed by MTT assay for cell viability and IC_50_ values (data not shown). To better observe autophagy-related features, subsequent in vitro experiments were performed by using chidamide at a concentration of 300 nM (which was much lower than its IC_50_ for each cell line), enabling a model wherein cell death fraction did not exceed 10%. As shown in Fig. 1a, b, chidamide treatment induced dose-dependent downregulation of LC3B at both mRNA and protein levels, but did not cause an observed increase in the ratio of LC3B-II to LC3B-I, referred to later as LC3 conversion. These data taken together suggest that chidamide markedly impedes LC3B expression, but does not have an impact on its lapidation. Again, chidamide treatment substantively blocked rapamycin-induced LC3B upregulation (Fig. 1c, d). Given that rapamycin is a standardized autophagy inducer, our results strongly suggest the autophagy-suppressive role of chidamide in MM cells. As is consistent with these findings, electron microscopic studies revealed that rapamycin was able to stimulate myeloma cells to generate numerous autophagic vesicles, whereas chidamide-treated or untreated cells displayed few such attributes (Fig. 1e). Collectively, these data suggest that chidamide not only disrupts the formation of autophagosomes, but also represses expression of LC3B in MM cells.

### Chidamide results in global upregulations of H4K16ac and H3K27me3 histone marks

**Fig. 2:**
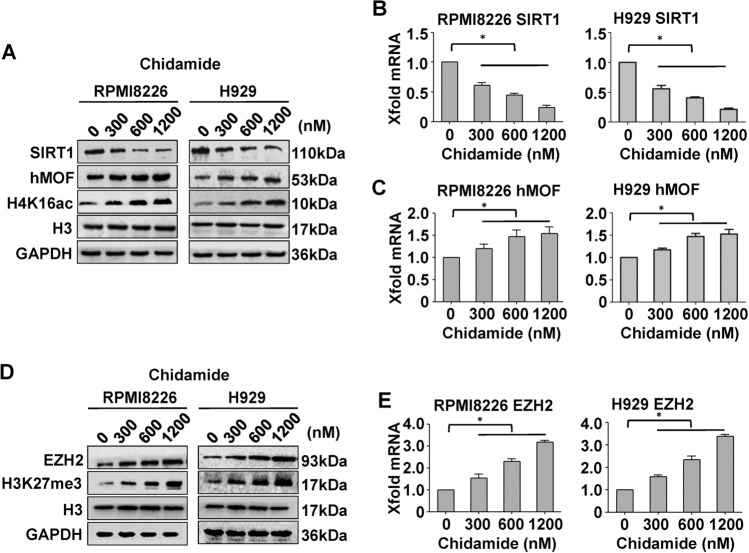
Effects of chidamide on expressions of histone-modifying enzymes (hMOF, SIRT1, and EZH2), and amounts of histone modifications (H4K16ac and H3K27me3) in MM cells. RPMI8226 and H929 cells were treated for 48 h with chidamide at the indicated concentrations. **a**, **d** The protein levels of SIRT1 (**a**), hMOF (**b**), or EZH2 (**d**) and amounts of H4K16ac (**a**) or H3K27me3 (**d**) were determined by immunoblotting with the indicated antibodies. Both H3 and GAPDH were used as controls for protein loading. Blots shown are representative of three independent experiments. **b**, **c**, **e** Relative mRNA levels of SIRT1 (**b**), hMOF (**c**), and EZH2 (**e**) were detected by using quantitative RT-PCR. Mean ± SD of three independent experiments. *P < 0.05, compared with the treatment-naive control.

Histone modifications play a critical role in epigenetic regulation of autophagic gene transcription^40,41^. For improving on understanding the role for histone marks in chidamide-induced autophagy inhibition, we investigated the effects of chidamide on the global amounts of H4K16ac and H3K27me3, respectively. As shown in Fig. 2a, chidamide treatment caused dose-dependent upregulation of H4K16ac, but did not affect the total histone H3 amounts. As the equilibrium of hMOF and SIRT1 expression influences the acetylation status of H4K16^10^, we next determined the effect of chidamide on their expressions. As shown by immunoblotting and qRT-PCR analysis (Fig. 2a–c), chidamide treatment markedly downregulated SIRT1 and modestly upregulated hMOF at both mRNA and protein levels, thereby shifting the balance of hMOF and SIRT1 expression in favor of hMOF and enabling the upregulation of H4K16 acetylation. Simultaneously, chidamide treatment also upregulated EZH2 expressions (Fig. 2d, e), leading to a dose-dependent increase in H3K27 trimethylation (Fig. 2d). These data together suggest that chidamide treatment leads to global upregulations of H4K16ac and H3K27me3 histone modifications, at least in part, via modulating the expressions of chromatin-modifying enzymes (such as hMOF, SIRT1, and EZH2).

### Chidamide specifically increases amounts of H4K16ac and H3K27me3 at the LC3B promoter

**Fig. 3:**
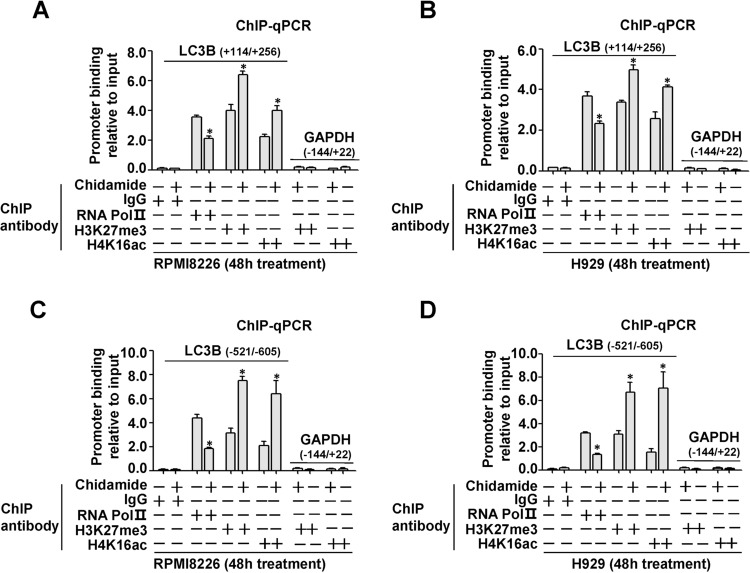
Effects of chidamide on amounts of H4K16ac and H3K27me3 histone marks at LC3B promoter regions in MM cells. RPMI8226 and H929 cells were treated for 48 h with chidamide (300 nM), and then subjected to chromatin immunoprecipitation (ChIP) analysis with the indicated antibodies (IgG served as a negative control). Occupancy of RNA Pol II, H4K16ac, and H3K27me3 in the LC3B promoter regions +114/+256 (**a**, **b**) or −521/−605 (**c**, **d**) was measured by quantitative PCR and expressed as fold enrichment relative to the input chromatin. Mean ± SD of three independent experiments. *P < 0.05, compared with the untreated control. Nontarget gene GAPDH promoter region –144/+22 was used as a PCR-negative control.

As indicated above, chidamide treatment significantly upregulated H4K16ac and H3K27me3 histone modifications at global levels (Fig. 2a, d), and concurrently led to marked reduction in LC3B mRNA levels (Fig. 1a). To address the possibility that LC3B represents a transcriptional target of H4K16ac and H3K27me3 histone marks, ChIP coupled to qPCR was performed to determine whether H4K16ac and H3K27me3 could be detected at the LC3B promoter. To this end, we mapped the corresponding LC3B promoter regions for specific quantification of H4K16ac and H3K27me3. As seen in Fig. 3a–d, we observed no PCR signals from negative controls (anti-rabbit IgG) and from GAPDH promoter region −144/+22 (PCR-negative controls), which exhibited neither nonspecific precipitation nor PCR contamination. In contrast, the positive signals were found in the samples treated with H4K16ac or H3K27me3 antibody, which suggest specific upregulation of H4K16ac or H3K27me3 at the LC3B promoter. More importantly, compared with untreated controls, chidamide treatment resulted in significant and concurrent increase in H4K16ac and H3K27me3 accompanying reduction in RNA polymerase II binding at the corresponding promoter regions. Furthermore, the specific ChIP signals that were detected in the region −521/−605 were more than those in the region +114/+256. Taken together, these findings demonstrate that both H4K16ac and H3K27me3 are specifically and concomitantly increased at the promoter regions around the transcription start site, thereby mediating chidamide-induced LC3B mRNA downregulation.

### Chidamide and botezomib induce synergistic cytotoxicity against MM cells

**Fig. 4:**
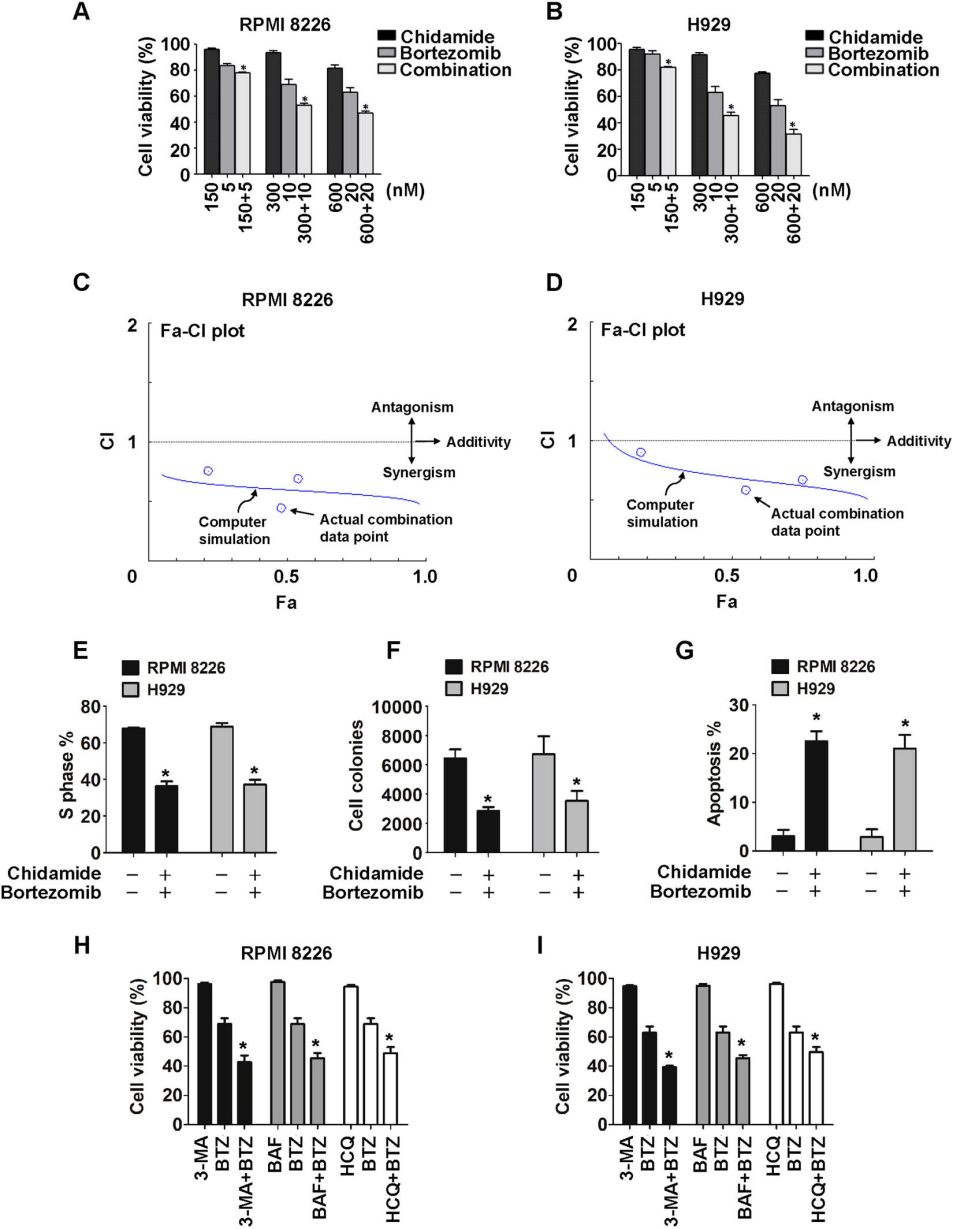
Chidamide plus bortezomib induce synergistic cytotoxicity in MM cells. **a**, **b** RPMI8226 (**a**) and H929 (**b**) cells were treated for 48 h with the indicated concentrations of chidamide and bortezomib, either alone or in combination, and then analyzed for viability by using an MTT assay. Mean ± SD of three independent experiments. *P < 0.05 versus the single-agent groups. **c**, **d** Fa–CI plots were calculated based on the Chou–Talalay equation using CompuSyn software version 1.0. Each round symbol designated the CI (combination index) values for each Fa (fraction affected) at three different dose points. **e**–**g** MM cells were treated for 48 h with bortezomib (10 nM) plus chidamide (300 nM). The cells in the S phase (**e**) were measured by flow cytometry using BrdU–FITC staining; the colony-formation potential (**f**) was detected by soft-agar clonogenic assay; the apoptotic cells (**g**) were determined by flow cytometry using annexin V–FITC/PI staining. Mean ± SD of three independent experiments. *P < 0.05, compared with the untreated group. **h**, **i** RPMI8226 and H929 cells were treated for 48 h with bortezomib (10 nM) in the presence or absence of hydroxychloroquine (HCQ, 3 µM and 5 µM, respectively), bafilamycin A1 (BAF, 1 and 3 nM, respectively), or 3-methyladenine (3-MA, 1.5 and 1 mM, respectively). Cell viability was determined as described above.

To determine the clinical implication of chidamide-induced autophagy suppression, we next evaluate the antimyeloma effect of dual-pathway inhibition, because concurrent targeting of UPS and autophagy may provide more effective treatment of MM^15^. To this end, RPMI8226 or H929 cells were treated with different doses of chidamide and bortezomib in monotherapy or in combination. As shown in MTT assays (Fig. 4a, b), the co-treatments resulted in enhanced cytotoxic effects on myeloma cells compared with each single agent. We subsequently analyzed the potency of drug combinations through CompuSyn^42–44^. As a result, the CI values generated in three different combination points were all less than 1 (data not shown), indicating synergistic results for all the doses used. Furthermore, as shown in Fa–CI plots (Fig. 4c, d), the combination of 300 nM chidamide and 10 nM bortezomib exhibited more potent synergistic cytotoxicity against two cell lines (as indicated by CIs of 0.46 and 0.59, respectively), revealing the optimal dose points for drug combination.

To further unveil the underlying mechanisms contributing to synergistic cytotoxicity, we investigate the effects of this optimal drug combination on colony formation and DNA synthesis in MM cells. As revealed by BrdU uptake assay (Fig. 4e) and soft-agar culture (Fig. 4f), the co-treatments significantly reduced the cells in the S phase and the number of colonies by 32–35% and 45–48%, respectively. Again, co-treatments led to 19–21% increase in cells with annexin V^+^ (Fig. 4g). Collectively, these data suggest that the combination of 300 nM chidamide and 10 nM bortezomib not only modestly induces apoptosis but also markedly inhibits proliferation, which jointly results in potent synergistic cytotoxicity against MM cells. Given that standardized autophagy inhibitors (such as hydroxychloroquine, bafilamycin A1, or 3-methyladenine) markedly potentiated antimyeloma activity by bortezomib (Fig. 4h, i), these data also suggest a critical role for chidamide-induced autophagy inhibition in contributing to synergistic cytotoxicity against MM cells.

### Chidamide and botezomib cooperatively induce antimyeloma activity by disrupting UPR signaling

**Fig. 5:**
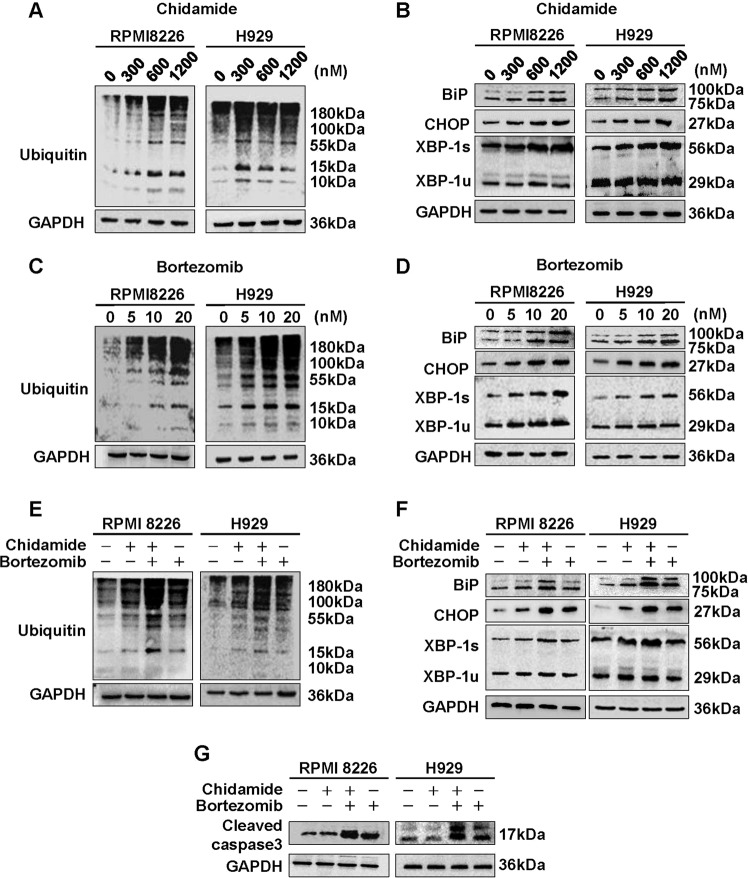
Effects of chidamide plus bortezomib on degradation of ubiquitinated proteins and expression of UPR-related components or proapoptotic protein in MM cells. RPMI8226 and H929 cells were treated for 48 h with chidamide (**a**, **b**) or bortezomib (**c**, **d**) at the indicated concentrations or with 10 nM bortezomib in the presence or absence of 300 nM chidamide (**e**–**g**). Protein lysates were subjected to immunoblotting with the indicated antibodies. GAPDH was used as a control for protein loading. Blots shown are representative of three independent experiments.

**Fig. 6:**
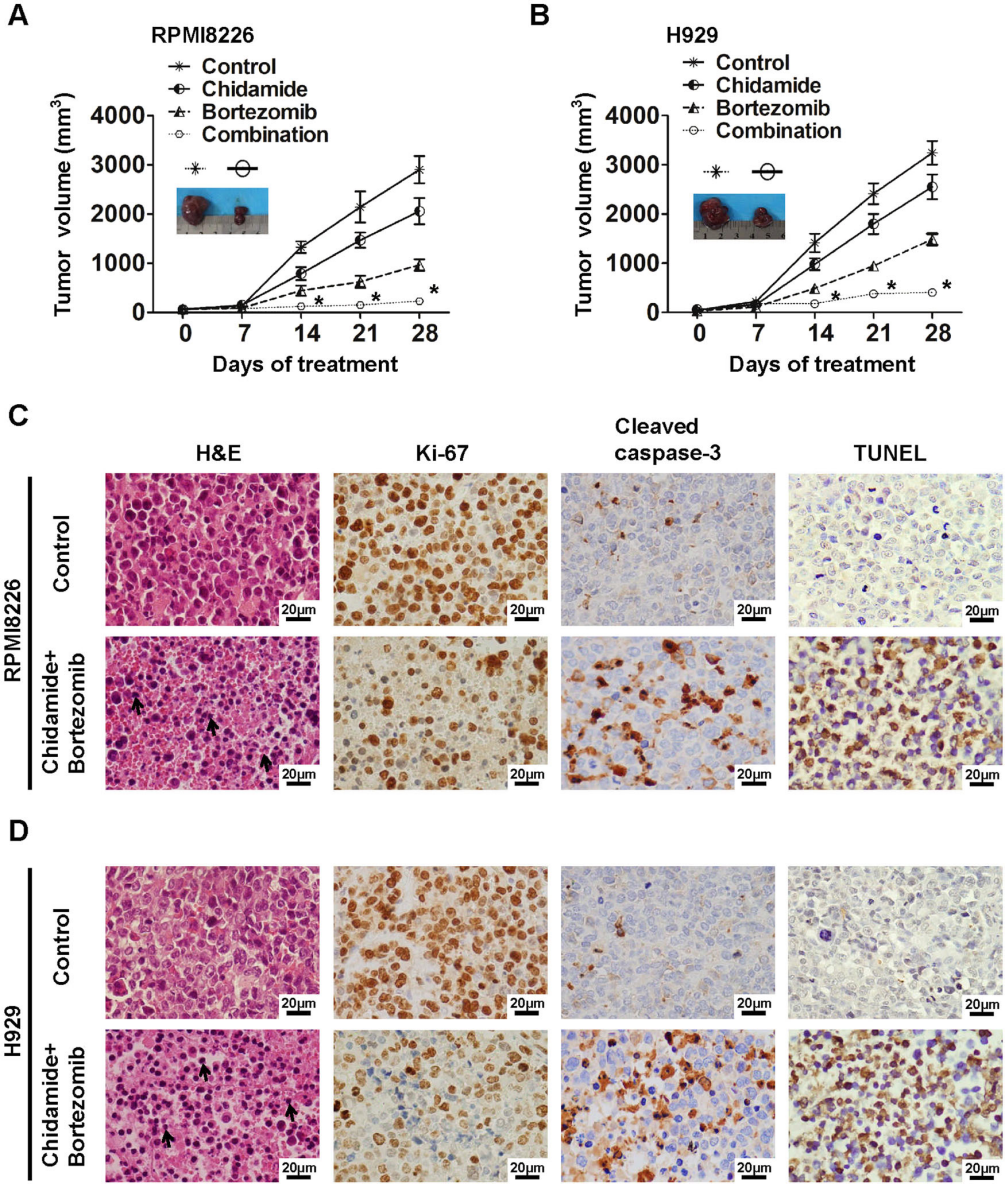
Chidamide plus bortezomib induce cooperative antimyeloma activities in plasmacytoma xenograft models. **a**, **b** RPMI8226 (**a**) and H929 (**b**) tumors were established subcutaneously and treated intraperitoneally with triweekly injections of chidamide (10 mg/kg) for 3 consecutive weeks, intravenously with biweekly injections of bortezomib (0.5 mg/kg) for 3 consecutive weeks, or their combination with the same dosing regimen used for the individual drugs. Tumor growth was monitored. Mean ± SD (*n* = 5). *P < 0.05, compared with PBS controls. Insets show tumors resected from mice 28 days after treatment with PBS or chidamide plus bortezomib. **c**, **d** The micrographs show apoptotic and proliferative cells in tumors sectioned on day 28 (endpoint) from the mice under the treatment regimens given. The apoptotic or proliferative cells were identified by a TUNEL assay as well as H&E and immunochemical staining. Dark-brown color indicates TUNEL- or Ki-67- or cleaved caspase-3-positive cells. Chromatin condensation is denoted by arrows. Scale bars: 20 μm. Original magnification, ×400.

Proteostasis placed on the ER depends upon UPS- and autophagy-mediated proteolysis, and is indispensable to the survival of MM cells^15^. For this reason, we next explore the effects of the optimal drug combination on proteotoxic stress of ER and adaptive UPR signaling. As shown in Fig. 5a–d, treatment with either chidamide or bortezomib markedly induced accumulation of polyubiquitinated proteins, and simultaneously increased UPR-related components (i.e., BiP, CHOP, and XBP-1) in a dose-dependent manner. Moreover, the ubiquitinated proteins, UPR components, and proapoptotic protein (i.e., cleaved caspase-3) were induced more by chidamide and bortezomib co-treatment than each agent alone (Fig. 5e–g). These findings are in consistent with the above results showing that co-treatment modestly induced apoptosis, but markedly reduced BrdU uptake and colony formation (Fig. 4e–g), and thus reinforce the crucial roles for both UPS and autophagy in degrading ubiquitinated proteins and regulating survival and proliferation in MM cells. Paralleling these data, similar antitumor efficacy also occurs in human plasmacytoma xenograft mouse model (Fig. 6a–d), wherein treatment with chidamide plus bortezomib led to a significant (75–80%) reduction in tumor growth relative to untreated mice (Fig. 6a, b, insets). To further understand the underlying mechanisms crippling tumor growth, both apoptotic and proliferative biomarkers were examined by using TUNEL assay and immunochemical staining. As revealed in paraffin-embedded sections of xenografted tumors treated with chidamide/bortezomib combinations (Fig. 6c, d), the apoptotic cells (i.e., TUNEL- and cleaved caspase-3-positive cells) were modestly increased as is consistent with the morphologic features of apoptosis (such as chromatin condensation). By contrast, the proliferative cells (i.e., Ki-67-positive cells) were markedly reduced. Collectively, these data imply that chidamide and bortezomib may act cooperatively in inducing excessive ER stress or perturbed UPR signaling, thereby triggering proteotoxic stress-initiated antimyeloma activity through apoptosis induction as well as proliferation inhibition.

## Discussion

Post-translational histone modifications (such as methylation and acetylation) influence chromatin structure and function, resulting in the various outcomes of cellular processes including autophagy^40^. Accordingly, our results demonstrated that the novel HDAC inhibitor chidamide pronounced an inhibitory effect on autophagosome formation and LC3B gene expression. These data promote us to further identify the role of epigenetic mechanisms in chidamide-induced autophagy inhibition in myeloma cells. Consequently, we observed that chidamide treatment robustly increased the global amount of H4K16ac by switching the equilibrium of hMOF and SIRT1 expression toward hMOF. Of note, chidamide-elicited SIRT1 downregulation was more pronounced than hMOF upregulation, implying that chidamide predominantly modulates SIRT1 expression (although the underlying molecular basis for this phenomenon is still to be elucidated). Recently, the global amount of H4K16ac was shown to decrease during rapamycin-induced autophagy, wherein H4K16 deacetylation was coupled predominantly to the downregulation of ATG genes^8^. However, inconsistent with this report, our data revealed that an increased amount of H4K16ac was linked to transcriptional repression of LC3B. Our data indicate that the role of H4K16 acetylation in regulating the autophagy program appears more complex than hitherto believed, which may depend upon context, stimulation, and cell-type specificities. We thus speculate that other mechanisms (in addition to the H4K16ac-associated scenario) may cooperatively contribute to epigenetic regulation of chidamide-induced autophagy inhibition. As expected, we observed increased expression of EZH2 and the associated upregulation of H3K27 trimethylation at global levels in chidamide-treated MM cells, thereby implying that EZH2-mediated upregulation of H3K27me3 is required for chidamide-induced autophagy suppression. These data further support the conclusion that EZH2 inhibition-mediated downregulation of H3K27me3 effectively activates the autophagy program in cancer cells^4,6^. Altogether, our findings reinforce the pivotal roles for H4K16 acetylation and H3K27 trimethylation in chidamide-induced autophagy inhibition in MM cells.

As mentioned above, global increases in H4K16ac and H3K27me3 were associated with chidamide-induced downregulation of LC3B mRNA. We therefore investigated the specific roles for H4K16ac and H3K27me3 in epigenetic regulation of LC3B transcription subsequent to chidamide treatment. As a result, we observed concomitant and pronounced increases in H4K16ac and H3K27me3 in the same regions of the LC3B promoter. These findings reveal the specific and crucial roles for the coexistence of H4K16ac and H3K27me3 in mediating transcriptional repression of the LC3B gene, further strengthening the assertion that H4K16 acetylation and H3K27 trimethylation influence higher-order chromatin configuration and have important roles in gene transcription^41^. The current data also suggest that the elaborate coordination between H4K16ac and H3K27me3 may serve as a part of the global mechanisms by which chidamide represses LC3-dependent autophagy, although the exact cooperative mechanism remains to be elucidated. Nonetheless, our data are in parallel with the concept that multiple molecular interplays between the enzymes that install histone marks constitute a highly sophisticated system associated with epigenetic regulation of transcription^1,2^.

Myeloma cells are uniquely reliant on cellular proteostasis for their survival. Targeting this vulnerability with proteasome inhibitor bortezomib has proved successful for treatment of MM^17–19,21,22^. More importantly, deficiency in UPS robustly facilitates autophagic degradation^15,16,20^, which is largely presumed to represent a compensatory response to compromised removal of ubiquitinated proteins^15,16,20^. This interaction between UPS and autophagy promotes and guides us to further investigate the antimyeloma action of dual-pathway suppression. Based on the powerful synergy of 300 nM chidamide and 10 nM bortezomib, subsequent experiments were performed by using both drugs at the respective doses as above. Our results provided compelling evidence that concurrent inhibition of autophagic and proteasomal activities via co-treatment with chidamide and bortezomib markedly blocked ubiquitinated protein degradation and resulted in persistent ER stress or perturbed UPR signaling, which thus led to modest apoptosis and marked growth arrest in myeloma cells. These findings are concurrent with previous studies showing cooperative antimyeloma activity of HDAC inhibitor combined with bortezomib^33–35^, and further confirmed by using human plasmacytoma xenograft models. Taken together, our results mechanistically suggest that excessive proteotoxic stress may, at least in part, constitute the underlying biochemical basis for in vivo and in vitro antimyeloma activities of chidamide plus bortezomib.

In conclusion, our results show that the cooperation and interplay between H4K16ac and H3K27me3 histone marks are involved in epigenetic regulation of chidamide-induced repression of the autophagy program in myeloma cells. Our results also unveil a regulatory network wherein autophagy deficiency-initiated excessive ER stress or dysregulated UPR signaling contributes to cooperative antimyeloma activity by chidamide combined with bortezomib. Importantly, these data provide a platform for future investigations to explore the implication of dual-pathway suppression for the treatment of MM.

## Supplementary information

Declaration of Contributions

Supplementary information

Supplementary figure 1

Supplementary figure 2

Supplementary figure 3

Supplementary figure 4

Supplementary figure 5
