# Supplementary material for: Chidamide epigenetically represses autophagy and exerts cooperative antimyeloma activity with bortezomib
Source: Cell Death Dis. 2020 Apr 27;11(4):297. doi: 10.1038/s41419-020-2414-3 (PMC7186232; doi:10.1038/s41419-020-2414-3)
Supplement: Supplementary file 1 — Declaration of Contributions [file 41419_2020_2414_MOESM1_ESM.pdf]

DECLARATION OF CONTRIBUTIONS TO ARTICLE

ADMC

Manuscript Number: CDDIS-19-0730R

Journal Name: *Cell Death & Disease* (the 'Journal')

Proposed Title of the Contribution:

Chidamide epigenetically represses autophagy and exerts cooperative antimyeloma activity with bortezomib (the 'Contribution)

Author(s):

Li Xu, Juan Feng, Hailong Tang, Ying Dong, Mimi Shu, Xiequn Chen (the 'Authors')

For all *CDDis* articles, each person named as an author in the published version must be able to show he or she has contributed substantially to the article.

Authorship credit should be based on 1) substantial contributions to conception and design, acquisition of data, or analysis and interpretation of data; 2) drafting the article or revising it critically for important intellectual content; and 3) final approval of the version to be published. Authors should meet conditions 1, 2 and 3.

Any person who cannot be shown to have made a substantial contribution to the article cannot be listed as an author in the final version. The name of any person who is deemed to have made a minor contribution can, however, appear in the Acknowledgments section of the article.

Please complete the table below to indicate the contributions of all named authors to the manuscript.

Author Full Name:	Specification of Contribution to the Manuscript:
Li Xu	conception and design; acquisition of data; helping draft the article and revising it; final approval of the version to be published.
Juan Feng	conception and design; acquisition of data; helping draft the article and revising it; final approval of the version to be published.
Hailong Tang	conception and design; acquisition of data; helping draft the article and revising it; final approval of the version to be published.
Ying Dong	conception and design; acquisition of data; helping draft the article and revising it; final approval of the version to be published.
Mimi Shu	conception and design; analysis and interpretation of data; drafting the article and revising it; final approval of the version to be published.
Xiequn Chen	conception and design; designing all experiments; analysis and interpretation of data; drafting the article and revising it; final approval of the version to be published.

Please complete the table below to indicate the contributions of all named authors to the figures.

Figure 1:

Xiequn Chen and Mimi Shu analysed the data and contributed to the layout design. Li Xu generated the data and prepared panel A-E. Hailong Tang assembled the figure.

Figure 2:

Xiequn Chen and Mimi Shu analysed the data and contributed to the layout design. Li Xu and Hailong Tang generated the data and prepared panel A and D. Ying Dong generated the data and prepared panel B and C and E. Hailong Tang assembled the figure.

Figure 3:

Xiequn Chen and Mimi Shu analysed the data and contributed to the layout design. Li Xu and Ying Dong generated the data and prepared panel A-D. Hailong Tang assembled the figure.

Figure 4:

Xiequn Chen and Mimi Shu analysed the data and contributed to the layout design. Li Xu and Hailong Tang generated the data and prepared panel A-D. Juan Feng generated the data and prepared panel E-I. Hailong Tang assembled the figure.

Figure 5:

Xiequn Chen and Mimi Shu analysed the data and contributed to the layout design. Li Xu and Hailong Tang generated the data and prepared panel A-G. Hailong Tang assembled the figure.

Figure 6:

xiequn chen and mimi shu analysed the data and contributed to the layout design. Li Xu generated the data and prepared panel A and B. Juan Feng generated immunohistochemistry data, labelled the images, and prepared panel C and D. Hailong Tang assembled the figure.

Signed for and on behalf of the Author(s):	Print Name:	Date:
xiequn chen	Xiequn Chen	12th March, 2020
