# Supplementary figures and images for: Chidamide epigenetically represses autophagy and exerts cooperative antimyeloma activity with bortezomib
Source: Cell Death Dis. 2020 Apr 27;11(4):297. doi: 10.1038/s41419-020-2414-3 (PMC7186232; doi:10.1038/s41419-020-2414-3)

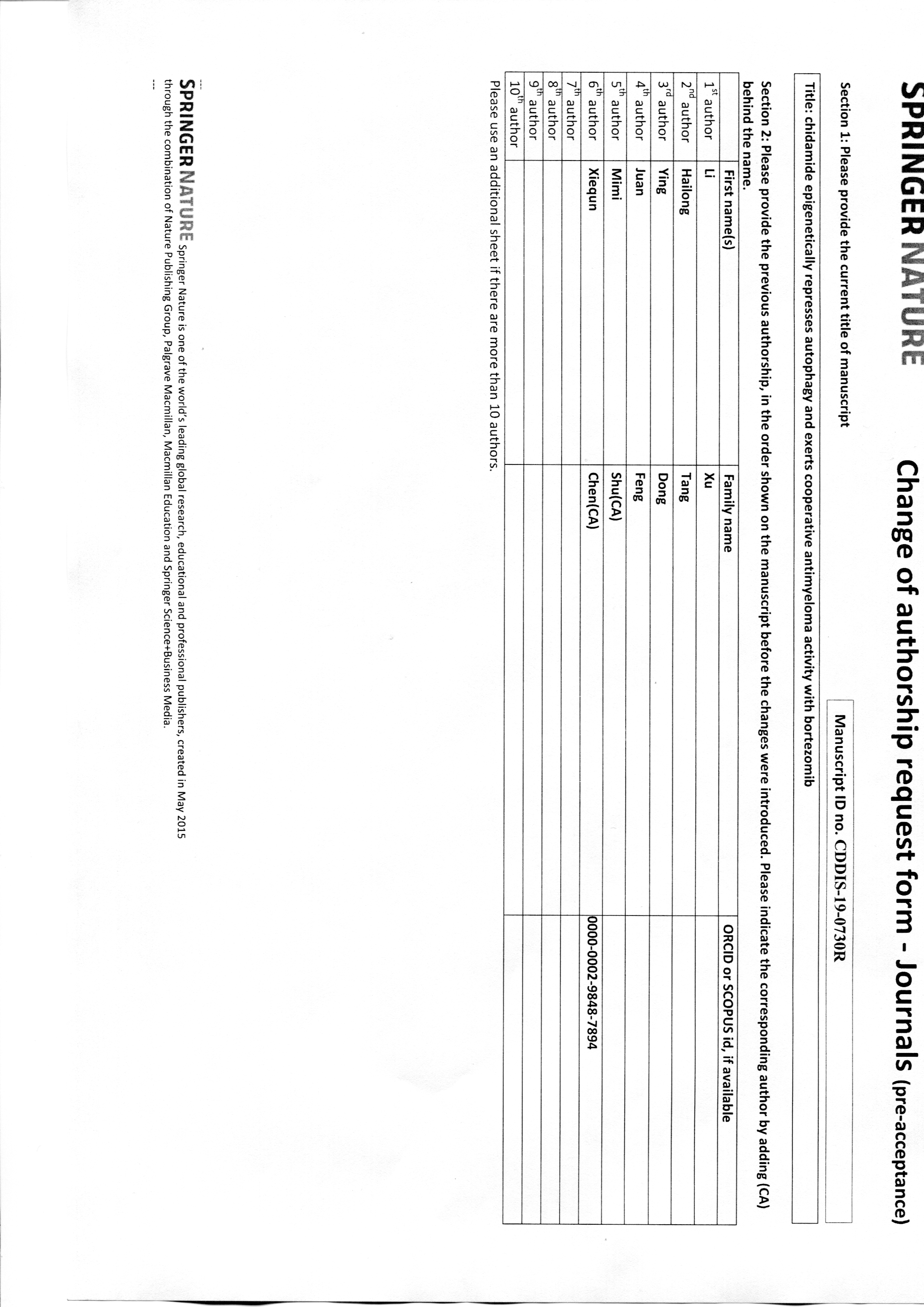

Supplement: Supplementary file 3 — Supplementary figure 1 [file 41419_2020_2414_MOESM3_ESM.tif]

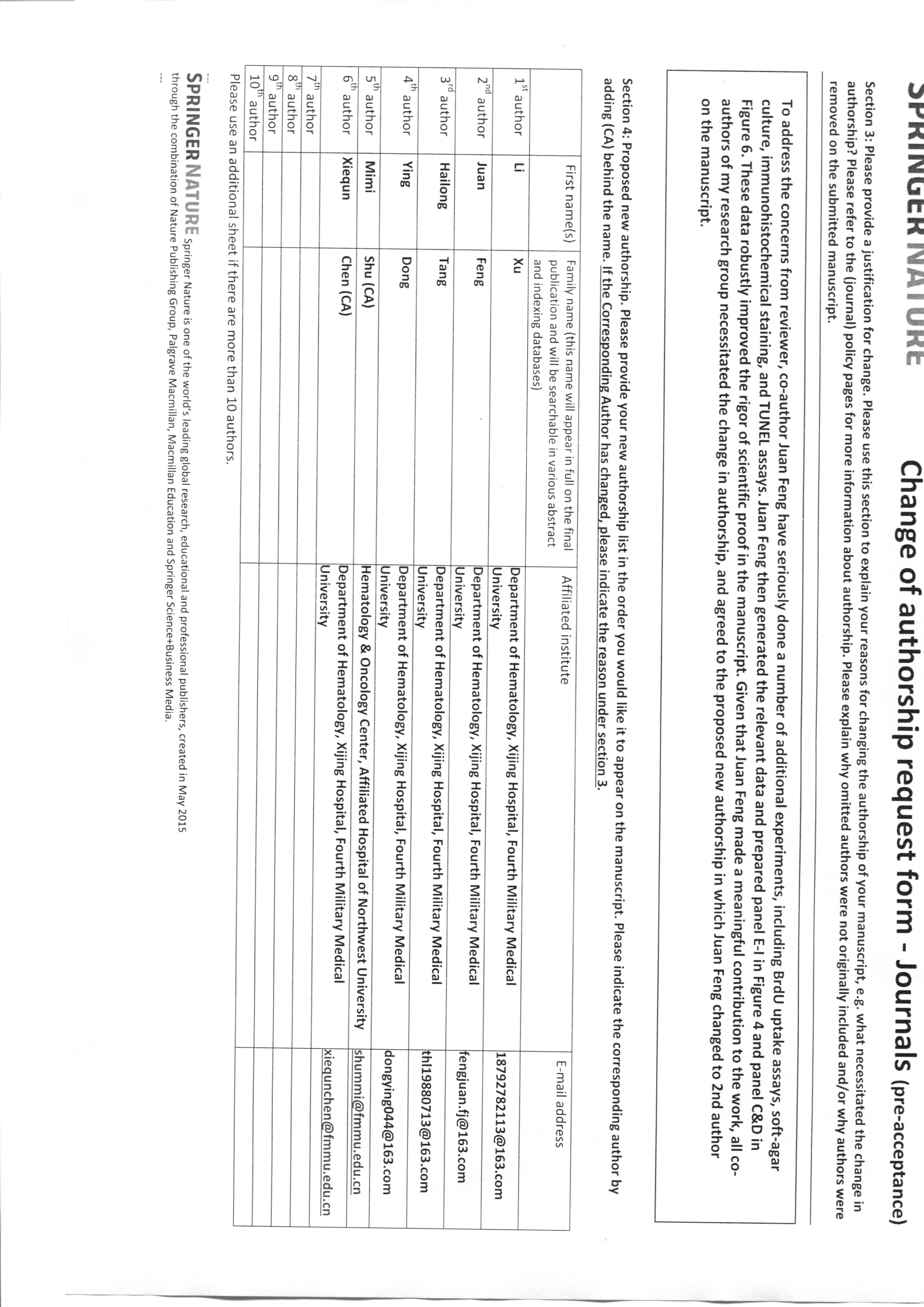

Supplement: Supplementary file 4 — Supplementary figure 2 [file 41419_2020_2414_MOESM4_ESM.tif]

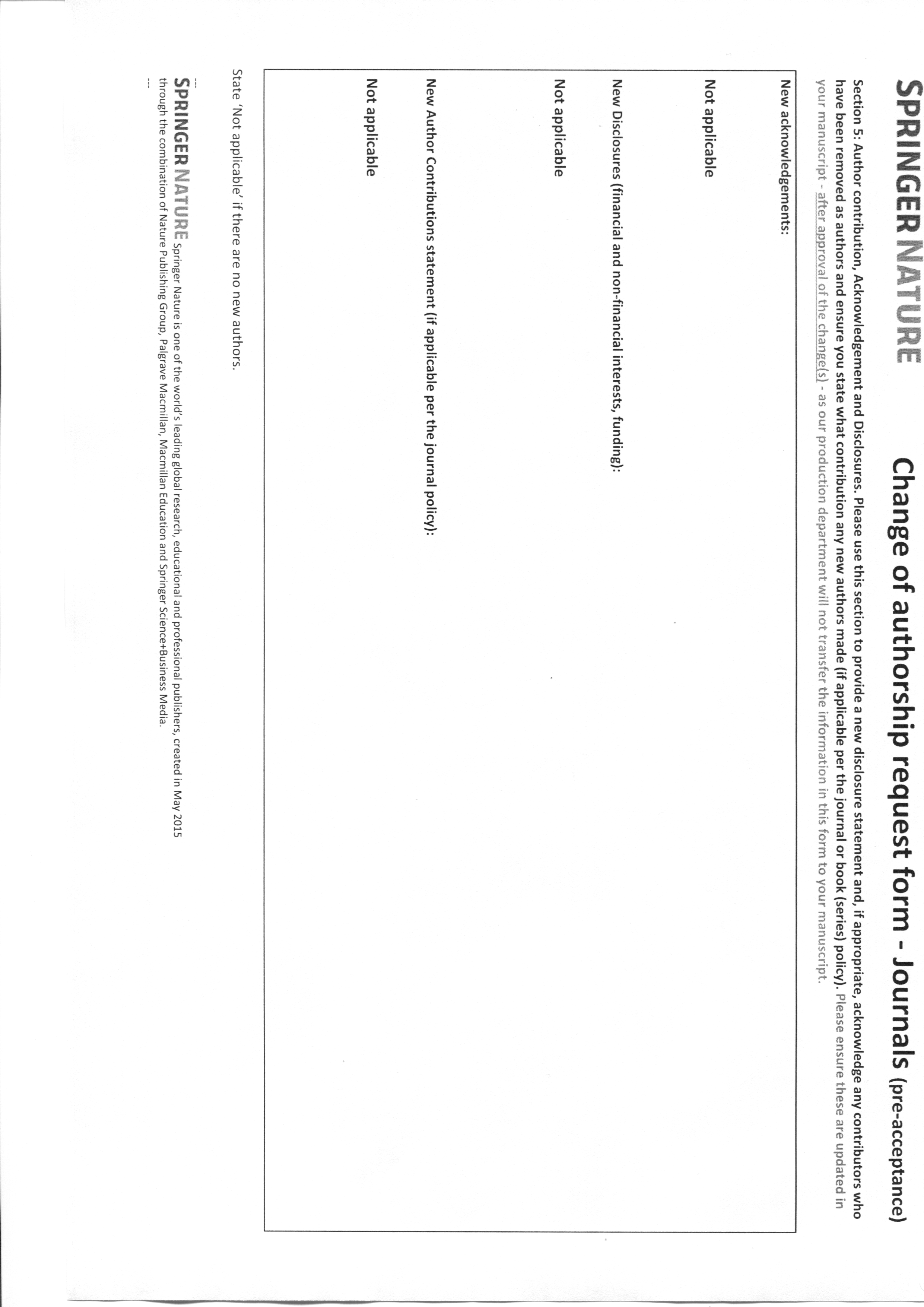

Supplement: Supplementary file 5 — Supplementary figure 3 [file 41419_2020_2414_MOESM5_ESM.tif]

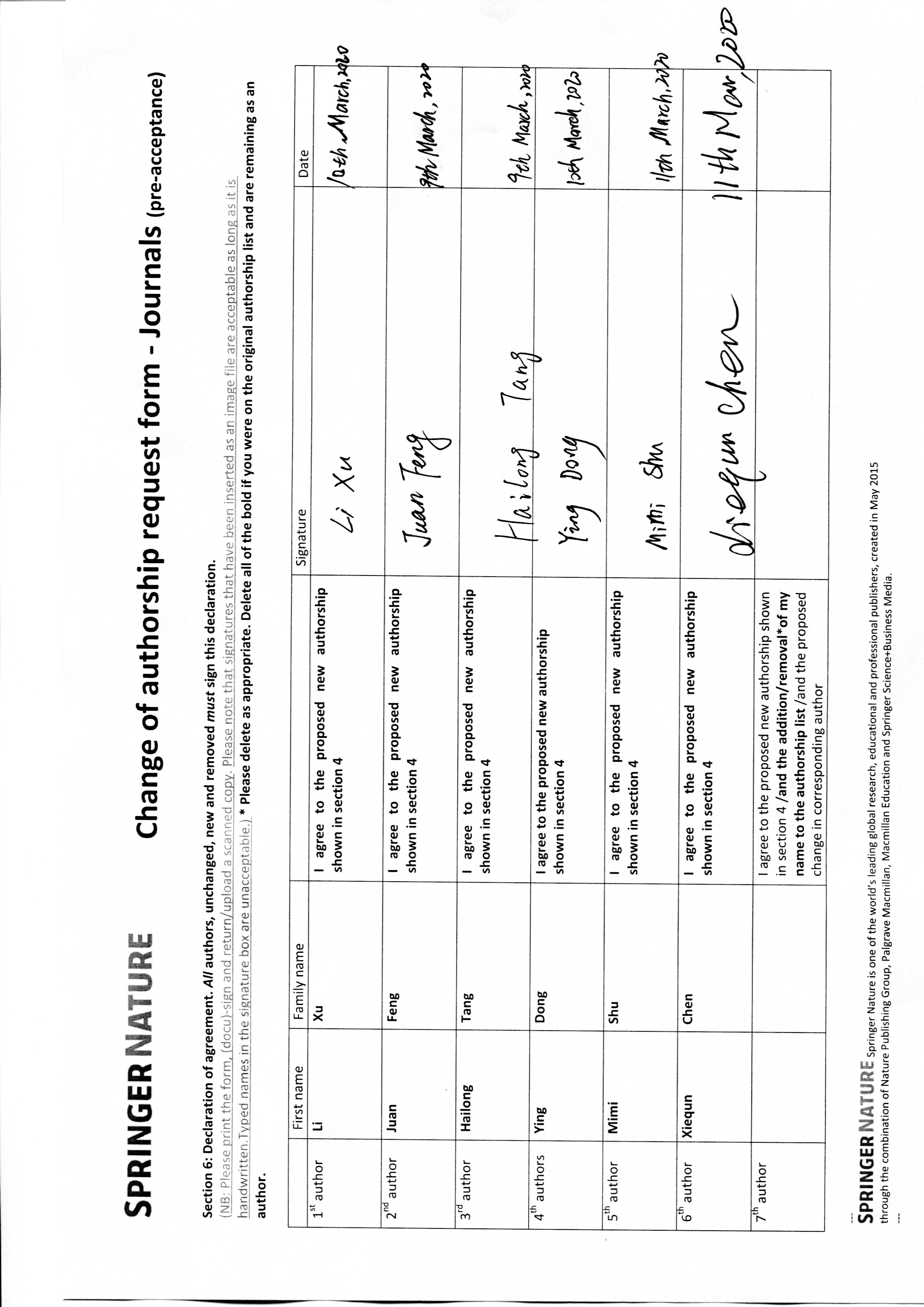

Supplement: Supplementary file 6 — Supplementary figure 4 [file 41419_2020_2414_MOESM6_ESM.tif]

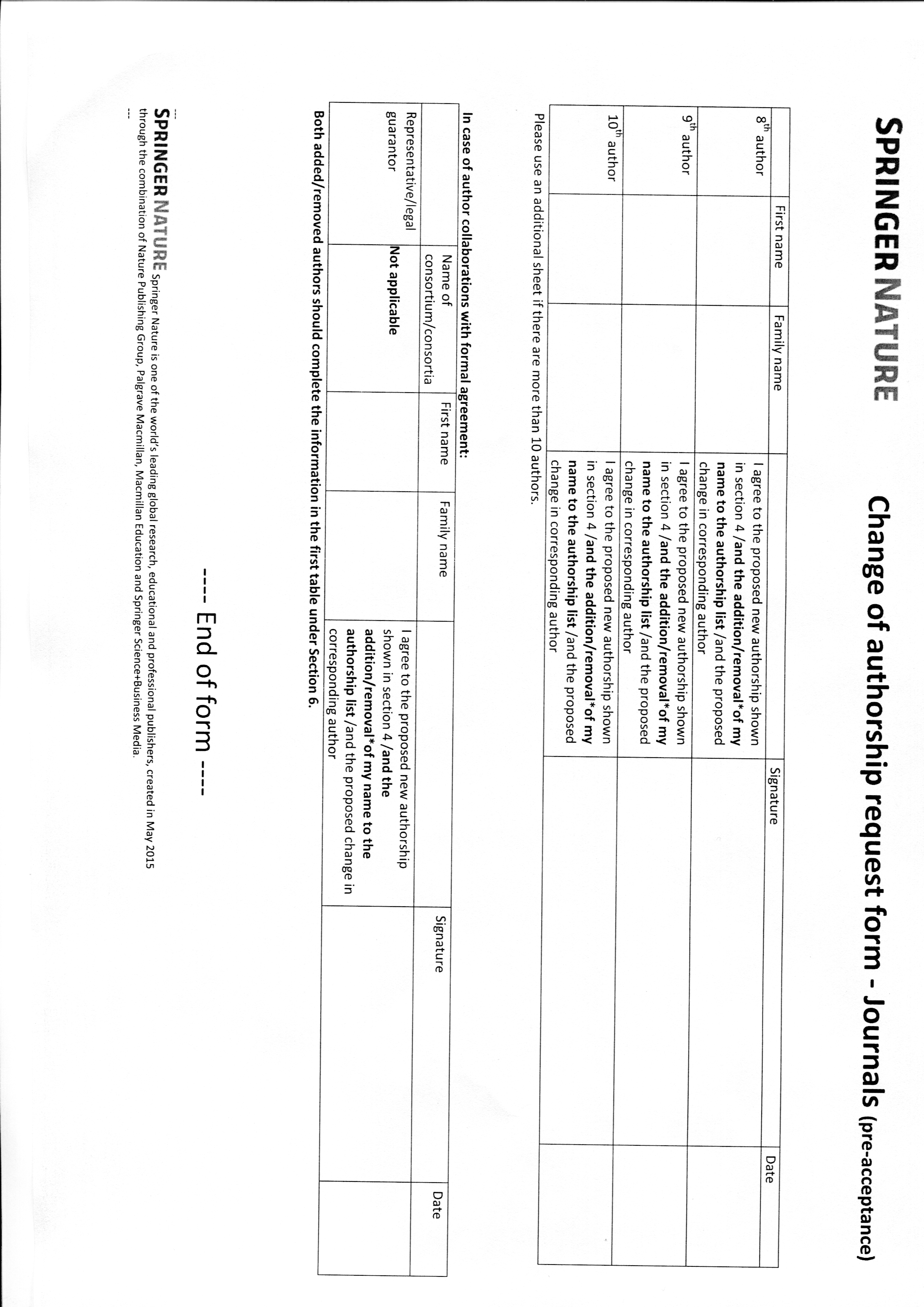

Supplement: Supplementary file 7 — Supplementary figure 5 [file 41419_2020_2414_MOESM7_ESM.tif]
